# Accelerated Global and Local Brain Aging Differentiate Cognitively Impaired From Cognitively Spared Patients With Schizophrenia

**DOI:** 10.3389/fpsyt.2022.913470

**Published:** 2022-06-22

**Authors:** Shalaila S. Haas, Ruiyang Ge, Nicole Sanford, Amirhossein Modabbernia, Abraham Reichenberg, Heather C. Whalley, René S. Kahn, Sophia Frangou

**Affiliations:** ^1^Department of Psychiatry, Icahn School of Medicine at Mount Sinai, New York City, NY, United States; ^2^Department of Psychiatry, Djavad Mowafaghian Centre for Brain Health, University of British Columbia, Vancouver, BC, Canada; ^3^Division of Psychiatry, University of Edinburgh, Edinburgh, United Kingdom

**Keywords:** cognitive subtypes, brainAGE, early psychosis, clustering, machine learning

## Abstract

**Background:**

Accelerated aging has been proposed as a mechanism underlying the clinical and cognitive presentation of schizophrenia. The current study extends the field by examining both global and regional patterns of brain aging in schizophrenia, as inferred from brain structural data, and their association with cognitive and psychotic symptoms.

**Methods:**

Global and local brain-age-gap-estimates (G-brainAGE and L-brainAGE) were computed using a U-Net Model from T_1_-weighted structural neuroimaging data from 84 patients (aged 16–35 years) with early-stage schizophrenia (illness duration <5 years) and 1,169 healthy individuals (aged 16–37 years). Multidomain cognitive data from the patient sample were submitted to Heterogeneity through Discriminative Analysis (HYDRA) to identify cognitive clusters.

**Results:**

HYDRA classified patients into a cognitively impaired cluster (*n* = 69) and a cognitively spared cluster (*n* = 15). Compared to healthy individuals, G-brainAGE was significantly higher in the cognitively impaired cluster (+11.08 years) who also showed widespread elevation in L-brainAGE, with the highest deviance observed in frontal and temporal regions. The cognitively spared cluster showed a moderate increase in G-brainAGE (+8.94 years), and higher L-brainAGE localized in the anterior cingulate cortex. Psychotic symptom severity in both clusters showed a positive but non-significant association with G-brainAGE.

**Discussion:**

Accelerated aging in schizophrenia can be detected at the early disease stages and appears more closely associated with cognitive dysfunction rather than clinical symptoms. Future studies replicating our findings in multi-site cohorts with larger numbers of participants are warranted.

## Introduction

Schizophrenia is a severe mental illness that presents with positive and negative symptoms and cognitive dysfunction (1). Cognitive abnormalities have been reported in multiple domains including executive function, processing speed, memory and attention (2). Additionally, schizophrenia is associated with structural abnormalities in brain regions that support these cognitive functions. These consist of reductions in the anterior cingulate, prefrontal and temporal cortical regions and in the volume of the hippocampus, amygdala, thalamus, and insula (3–6). Longitudinal studies suggest more rapid progressive decreases in brain tissue in patients with schizophrenia than in healthy individuals, primarily in frontal and temporal areas (7, 8) raising the possibility that processes related to schizophrenia may contribute to the premature development of age-related brain changes (i.e., accelerated aging). The notion of aberrant aging in schizophrenia is also supported by evidence of greater age-related cognitive decline (9), higher incidence of dementia (10), and biological markers of aging as indicated by shortened telomere length and accelerated epigenetic clocks (11–13).

The investigation of accelerated aging in schizophrenia benefits from developments in machine learning methods that enable the prediction of chronological age from neuroimaging data. The discrepancy between the neuroimaging-predicted age and the chronological age, termed the “brain-age-gap estimate” (brainAGE), is an individualized estimate of the degree of deviation from typical age-related brain changes (14, 15). A positive brainAGE indicates that the biological age of an individual's brain is “older” than their actual age, and a negative brainAGE reflects the inverse. In older populations, higher brainAGE has been associated with decline in executive function, memory, and information processing speed that are also directly relevant to schizophrenia (16).

Prior studies in schizophrenia have mainly used brain structural magnetic resonance imaging (sMRI) to compute brainAGE as a global measure that captures the totality of regional age-related changes (henceforth referred to as G-brainAGE). Multiple studies have reported elevated G-brainAGE in patients with schizophrenia (17–22); the largest such study reported an increase in G-brainAGE of about 4 years (Cohen's *d* effect size = 0.50) based on sMRI data from 2,598 healthy individuals and 2,803 patients with schizophrenia, aged 17–73 years (23). While these studies provide strong evidence for accelerated brain aging in schizophrenia, no study to date has examined the spatial variation of age-related changes which could identify brain regions that might be highly vulnerable. Additionally, the link between accelerated brain-aging and cognition remains largely unexplored despite its potential to provide mechanistic insights of therapeutic utility.

To address this gap, we applied a novel machine learning algorithm with a U-Net architecture (24) to sMRI data to compute G-brainAGE and voxel-level local brain-age (henceforth L-brainAGE) in patients with early-stage schizophrenia and healthy individuals. We used Heterogeneity through Discriminative Analysis (HYDRA) (25) to stratify patients based on their cognitive characteristics. Our working hypothesis was that accelerated aging, as inferred by G-brainAGE and L-brainAGE, will be greater in clusters of patients showing cognitive impairment and the highest deviance in terms of L-brainAGE will be observed in prefrontal and temporal regions where longitudinal changes have most consistently been reported in schizophrenia.

## Materials and Methods

### Samples

The data used in the current study were obtained from the 1.1 release of the Human Connectome Project Early Psychosis Study (HCP-EP; https://www.humanconnectome.org/study/human-connectome-project-for-early-psychosis/article/updated-hcp-early-psychosis-11-release) and the 1,200 subjects release of the Human Connectome Project Young Adults Study (HCP-YA; https://www.humanconnectome.org/study/hcp-young-adult) (details in Supplementary Material 1.1). These studies used harmonized protocols for sMRI data acquisition and cognitive assessment allowing data pooling. In the HCP-EP, the Structured Clinical Interview for DSM-5, Research Version (SCID-5-RV) (26) was used to establish the diagnostic status of patients and confirm illness onset within the past 5 years. The diagnostic status of healthy individuals was established using the SCID-5-RV in the HCP-EP and specific eligibility criteria in HCP-YA (details in Supplementary Material 1.1).

The study sample comprised 84 patients with schizophrenia (age range: 16–35 years; 30.20% female) and 1,169 healthy individuals (age range: 16–37 years; 53.3% female) (Supplementary Table 1). Data collection at the original recruitment sites complied with the ethical standards of national and institutional committees on human experimentation and with the Helsinki Declaration of 1975, as revised in 2008. The data used for the current analyses were de-identified and accessed through the National Data Archive.

### Procedures

#### Cognitive and Clinical Assessment

Cognitive functioning in HCP-EP and HCP-YA participants was assessed using the Penn Emotion Recognition Task (27) and the NIH Cognition Toolbox (28) which includes the following sub-tests: dimensional change card sort test, flanker inhibitory control and attention test, oral reading recognition test, picture vocabulary test, pattern comparison processing speed test, list sorting working memory test, and picture sequence memory test (details in Supplementary Table 2). In patients, the intelligence quotient (IQ) was additionally evaluated using the Wechsler Abbreviated Scale of Intelligence, Second Edition (WASI-II) (29), while clinical symptoms and social function were, respectively, assessed with the Positive and Negative Syndrome Scale (PANSS) (30) and the Mental Illness Research, Education, and Clinical Center version of the Global Assessment of Functioning scale (MIRECC-GAF) (31). Lifetime antipsychotic medication dosage was recorded as Chlorpromazine (CPZ) equivalents using the Gardner approach (32) and as months of cumulative exposure.

#### HYDRA Cognitive Clusters

We used HYDRA to identify subgroups of patients based on the cognitive test performance measures from the NIH Cognition Toolbox and Penn Emotion Recognition Task (Supplementary Table 2) with and sex and age were modeled as covariates. The WASI-II IQ was not used in clustering but was used for external cluster validation. HYDRA is a semi-supervised machine learning tool that clusters cases based on their differences from a healthy reference sample by finding multiple linear hyperplanes, which together form a convex polytope, thus extending linear max-margin classifiers to the non-linear space (https://github.com/evarol/HYDRA). We used 5-fold cross validation to determine the clustering stability for a range of 2–5 clusters based on the adjusted Rand index (ARI) (33); the solution with the highest ARI was chosen. Statistical significance was established with permutation testing compared to a null distribution (details in Supplementary Material 1.2). In order to depict the cognitive profiles of the clusters identified using HYDRA, we standardized performance on each cognitive test by z-score transforming scores based on normative means for the NIH Toolbox, and across all individuals included in the study for the Penn Emotion Recognition Task subtests. To test algorithm-independent reproducibility, the cognitive data were submitted to k-means clustering implemented in R using version 3.0 of the *NbClust* package (Supplementary Material 1.2).

#### Neuroimaging Acquisition and Preprocessing

Whole-brain T_1_-weighted data in the HCP-EP and HCP-YA samples were acquired on Siemens 3T scanners using similar 3D magnetization-prepared rapid gradient-echo (MPRAGE) sequences (details in Supplementary Material 1.3). All images were processed locally with the Statistical Parametric Mapping Toolbox (SPM12; https://www.fil.ion.ucl.ac.uk/spm/software/spm12/) using established procedures (details in Supplementary Material 1.3). Briefly, in each participant, T_1_-weighted images were normalized using affine followed by non-linear registration, corrected for bias field inhomogeneities, and segmented into gray and white matter, and cerebrospinal fluid. The Diffeomorphic Anatomic Registration Through Exponentiated Lie algebra algorithm (DARTEL) (34) was applied to normalize the segmented scans into a standard MNI space (MNI-152 space).

#### Computation of L-brainAGE and G-brainAGE

The gray and white matter outputs generated after preprocessing served as input to a convolutional neural network (U-Net) (https://github.com/SebastianPopescu/U-NET-for-LocalBrainAge-prediction) to yield voxel-wise estimates of L-brainAGE using parameters pre-trained in an independent sample of 4,155 healthy individuals aged 18–90 years (details in Supplementary Material 1.4). Importantly, the HCP-EP and HCP-YA datasets were not used in the development of the L-brainAGE model. The resulting L-brainAGE images provide information about voxel-level differences between neuroimaging-predicted age and chronological age for each individual. Subsequently, the G-brainAGE was computed in each participant by averaging voxel-level L-brainAGE information across the brain (details in Supplementary Material 1.4). To account for residual associations with age, G-brainAGE was corrected in all study participants by regressing out chronological age using established methods (35); unless otherwise specified, the corrected G-brainAGE values were used in all subsequent analyses while analyses involving L-brainAGE included age as a covariate. For both L-brainAGE and G-brainAGE, positive values indicate an older appearing brain than what would be predicted by chronological age. Model accuracy was ascertained using the voxel-level Mean Absolute Error (MAE), which quantifies the absolute difference between the neuroimaging-predicted age and the chronological age, and by the correlation between predicted age (averaged across voxels) and chronological age (unadjusted for chronological age).

### Statistical Approach

Statistical significance across all comparisons was set at *P*_FWE_ < 0.05 with family-wise-error correction. Case-control differences in L-brainAGE were identified using General Linear Models in SPM12 with age, sex, and site modeled as covariates. Case-control differences in G-brainAGE were assessed using analysis of covariance with group as a fixed factor, and sex and site included as covariates. Associations between clinical symptoms, social functioning, and WASI-II IQ with G-brainAGE and L-brainAGE were, respectively, assessed using Spearman's correlation and general linear models in SPM12. Follow-up sensitivity analyses in patients were conducted to test for the effects of medication dose.

## Results

### Cognitive Clusters in Patients

**Figure 1:**
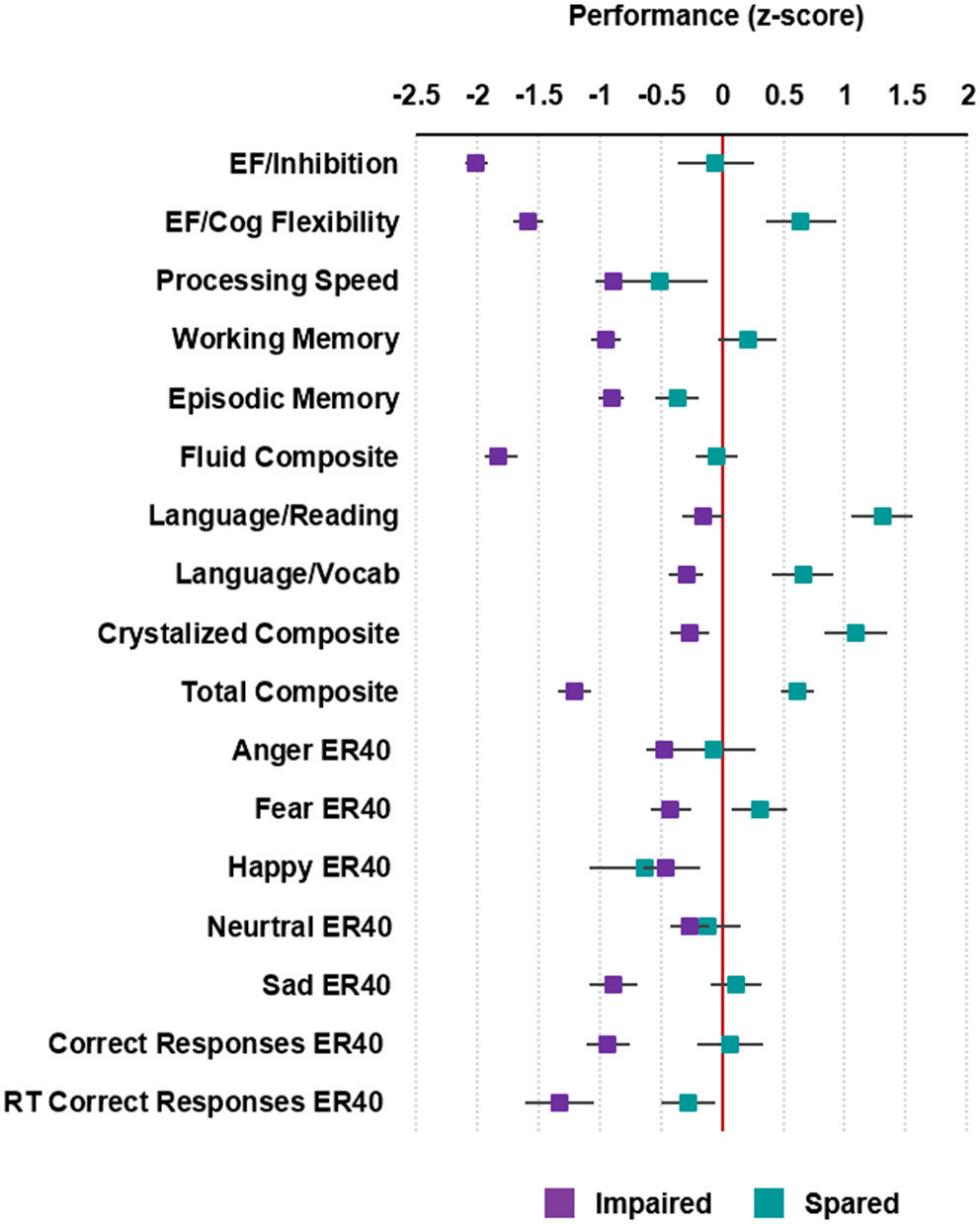
Cognitive profiles of the patients with schizophrenia. Average standardized cognitive performance profiles in the impaired and spared cognitive subgroups identified using HYDRA clustering. Error bars represent standard error. For RT Correct Responses ER40, the directionality of values was reversed so that higher values denote better performance. Executive Function/Inhibition: NIH Toolbox Flanker Inhibitory Control and Attention Test; Executive Function (EF)/Cognitive Flexibility: NIH Toolbox Dimensional Change Card Sort Test; Processing Speed: NIH Toolbox Pattern Comparison Processing Speed Test; Working Memory: NIH Toolbox List Sorting Working Memory Test; Episodic Memory: NIH Toolbox Picture Sequence Memory Test; Fluid IQ: NIH Toolbox Cognition Fluid Composite; Language/Reading: NIH Toolbox Oral Reading Recognition Test; Language/Vocabulary: NIH Toolbox Picture Vocabulary Test; Crystalized IQ: NIH Toolbox Cognition Crystallized Composite; Full IQ: NIH Toolbox Cognition Total Composite Score; Penn Emotion Recognition Test: Number of Correct Anger Identifications: Anger ER40; Penn Emotion Recognition Test: Number of Correct Fear Identifications: Fear ER40; Penn Emotion Recognition Test: Number of Correct Happy Identifications: Happy ER40; Penn Emotion Recognition Test: Number of Correct Neutral Identifications: Neutral ER40; Penn Emotion Recognition Test: Number of Correct Sad Identifications: Sad ER40; Penn Emotion Recognition Test Number of Correct Responses: Correct ER40: Penn Emotion Recognition Test: Correct Responses Median Response Time: RT Correct Responses ER40.

HYDRA identified two clusters of patients based on their cognitive profiles (Table 1; Figure 1). K-means clustering of the cognitive test performance measures yielded the same results (Supplementary Material 2.1; Supplementary Figure 1). The two HYDRA clusters did not differ in terms of age, sex, years of education, PANSS positive and negative symptoms, and MIRECC-GAF scores for occupational and social functioning, but patients in cluster 1 had received higher doses of antipsychotic medication (Table 1).

**Table 1:** Demographic and clinical characteristics of the cognitive subgroups of schizophrenia patients.

**Measure**	**Impaired** **(*N* = 69)**	**Spared** **(*N* = 15)**
**Sociodemographic**		
Age (years), mean (SD)	22.44 (3.41)	22.19 (3.65)
Female sex, *N* (%)	22 (31.9%)	4 (26.7%)
Years of education, mean (SD)	13.25 (1.67)	13.83 (2.98)
WASI-II IQ^**1a**^	96.90 (17.24)	109.93 (10.53)
**Clinical**		
Antipsychotic naïve, *N* (%)	3 (4.3%)	0 (0%)
Antipsychotic dose (CPZE), mean (SD)^**a**^	244.20 (247.12)	33.33 (104.65)
Antipsychotic drug exposure (months), mean (SD)	16.07 (15.34)	13.07 (14.11)
PANSS positive, mean (SD)	11.84 (3.83)	10.87 (4.47)
PANSS negative, mean (SD)	15.49 (6.02)	13.2 (4.62)
MIRECC-GAF occupational functioning	64.48 (23.82)	52.53 (23.49)
MIRECC-GAF social functioning	66.52 (15.82)	69.67 (14.53)

^1^*The WASI-II IQ was not included in the clustering*.

*^a^P_FWE_ = 0.05; all other P > 0.17 (uncorrected)*.

*CPZE, chlorpromazine equivalents; MIRECC-GAF, Mental Illness Research, Education, and Clinical Center version of the Global Assessment of Functioning scale; PANSS, Positive and Negative Syndrome Scale; SD, standard deviation; WASI-II, Wechsler Abbreviated Scale of Intelligence, Second Edition*.

Cluster 1 (*n* = 69) was considered “impaired” because the performance of patients assigned to this cluster was significantly lower compared to cluster 2 in all cognitive subtests of the NIH Toolbox (*P* < 0.05; Supplementary Table 3), with the exception of processing speed. Patients in cluster 1, also performed significantly worse in total correct responses (*T* = −2.56; *P* = 0.01) and identification of fearful (*T* = −2.00; *P* = 0.05) and sad faces (*T* = −2.36; *P* = 0.02) in the Penn Emotion Recognition Task. After z-score transforming cognitive performance in each of the cognitive subtests, patients in cluster 1 showed performance was decreased by 0.15–2.01 standard deviations (SD) compared to normative means (Figure 1). The greatest deviations in cognitive performance for individual subtests in cluster 1 were seen in executive function, both in inhibition (SD = −2.01) and cognitive flexibility (SD = −1.59). In contrast, cluster 2 (*n* = 15) was considered “spared” because the performance of patients assigned to this cluster in each of the cognitive subtests was generally within 0.5 SD of the normative means (Figure 1), with the exception of cognitive flexibility (SD = 0.64), reading decoding (SD = 1.31), vocabulary comprehension (SD = 0.66), and identification of happy faces (SD = −0.63). Confirming the validity of the clustering, the WASI-II IQ, which was not included as part of the clustering, was significantly lower in the impaired compared to the spared cluster (Cohen's *d* = −0.80, *P*_FWE_ < 0.05; Table 1).

### G-brainAGE in the Cognitively Impaired and Spared Clusters

The G-brainAGE model of healthy individuals had a MAE of 5.69 ± 3.57 (further details in Supplementary Material 2.2). The correlation between the chronological and predicted brain ages was *r* = 0.38 for healthy individuals. The impaired and spared clusters had numerically higher G-brainAGE [mean (SD): 11.08 (7.16) years and mean (SD): 8.94 (4.69) years respectively] compared to healthy individuals [mean (SD): 4.53 (4.73) years]. The age-corrected G-brainAGE was significantly higher in the cognitively impaired patient subgroup compared to healthy individuals (impaired: *F* = 29.11, *p* = 8.19E^−8^; Cohen's *d* = 0.58). The age-corrected G-brainAGE was comparable between healthy individuals and the cognitively spared patient subgroup (*F* = 2.39, *P* = 0.12; Cohen's *d* = 0.07). We did not observe any significant correlations between G-brainAGE with the PANSS positive or negative symptoms subscale scores, antipsychotic medication, MIRECC-GAF scores and WASI-II IQ within either cognitive cluster (Supplementary Table 4).

### L-brainAGE in the Cognitively Impaired and Spared Cluster

**Figure 2:**
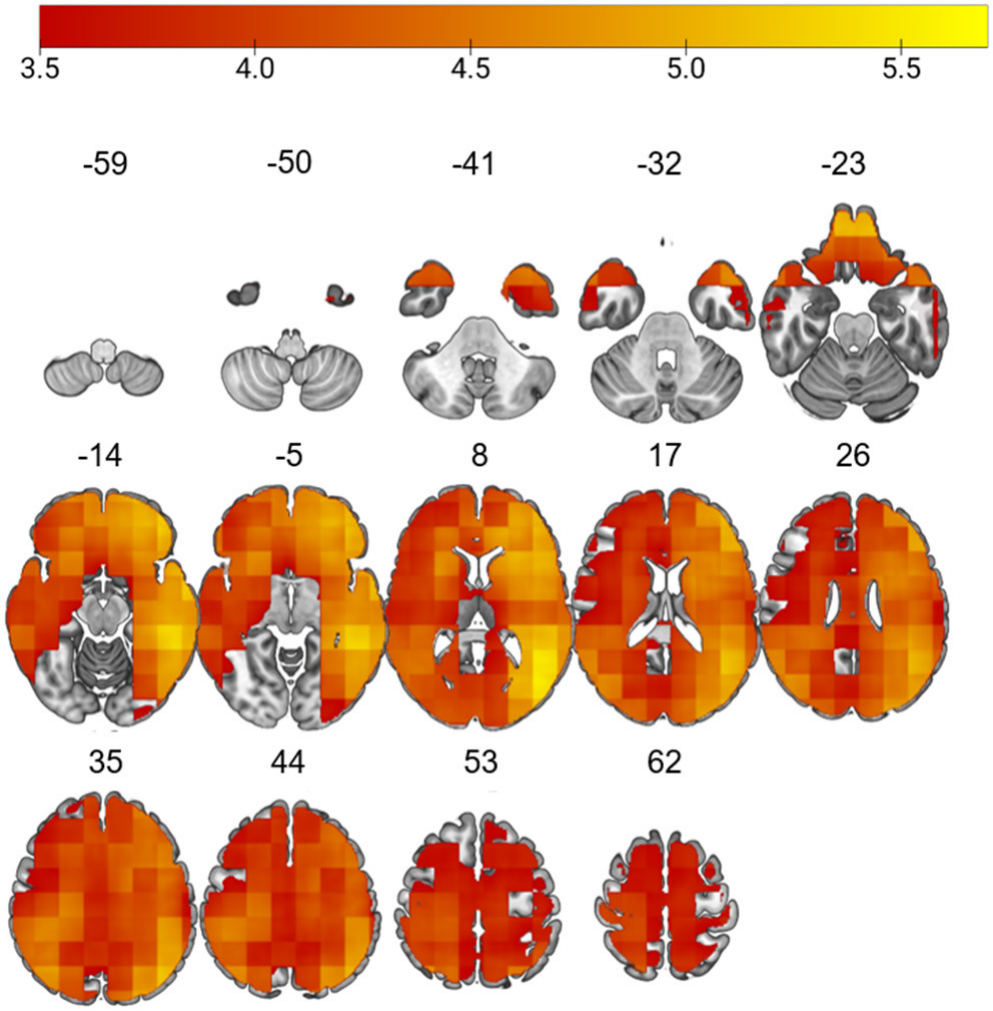
Differences in L-brainAGE between healthy individuals and the cognitively impaired patient subgroup. *T*-value overlay of statistically significant group differences in L-brainAGE based on a two-sample *t*-test comparing healthy individuals with the cognitive impaired patients (P_FWE_ < 0.05). The color bar shows regions in which patients with impaired cognition have higher brainAGE compared to healthy individuals. Images are displayed in neurological orientation with MNI coordinates.

**Figure 3:**
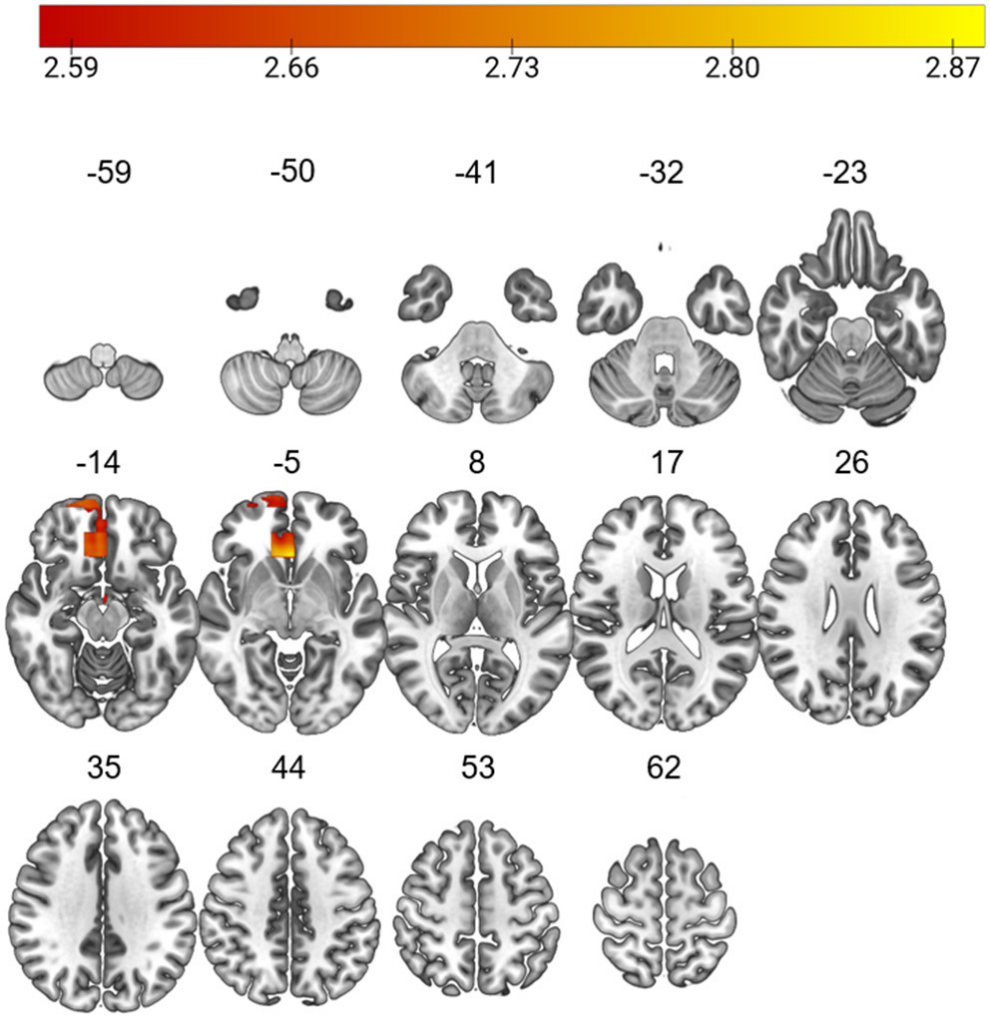
Differences in L-brainAGE between healthy individuals and the cognitively spared patient subgroup. *T*-value overlay of statistically significant group differences in L-brainAGE based on a two-sample *t*-test comparing healthy individuals with the cognitive spared patients (P_uncorrected_ < 0.005). The color bar shows regions in which patients with spared cognition have higher brainAGE compared to healthy individuals. Images are displayed in neurological orientation with MNI coordinates.

The L-brainAGE model yielded MAE ranging from 3.36 to 16.67 across voxels (further details in Supplementary Material 2.2; Supplementary Figures 2, 3). A general linear model with age, sex and site as covariates showed widespread increases in L-brainAGE in the impaired cluster compared to healthy individuals with the highest values observed in the middle and inferior temporal lobe, superior and middle frontal lobe, precuneus, and postcentral gyrus (*P*_FWE_ < 0.05; Figure 2). By contrast, comparison of the spared cluster to healthy individuals revealed a localized increase in L-brainAGE in the anterior cingulate cortex that was only observed at an uncorrected level of *P* < 0.005 (Figure 3). There was no significant association with L-brainAGE and current antipsychotic medication dose in CPZE, even at an uncorrected *P* < 0.05.

## Discussion

This is the first study to examine the regional pattern of accelerated brain aging in relation to profiles of cognitive impairment in patients with schizophrenia at the early stages of the disorder. The results show that patients with psychotic symptoms and cognitive underperformance evidence global and widespread local accelerated brain aging, while patients with psychotic symptoms and preserved cognitive functioning showed no differences in global accelerated aging and only minimal age-related acceleration in the anterior cingulate at the local level.

Application of machine learning to cognitive task performance data separated the current patient sample into a cognitively impaired (80%) and a cognitively spared (20%) cluster. Studies in patients with schizophrenia have consistently identified a cognitively spared cluster while cognitively impaired patients have been found to form up to 3 sub-clusters based on the degree of impairment (36–39). The current study is mostly closely aligned with that of Wenzel and colleagues (40), who also examined patients with schizophrenia within 2 years of the onset of psychosis. Patient stratification was based on their performance in tasks of attention, processing speed, executive function and memory and yielded an impaired cluster characterized by underperformance across all tasks and a spared cluster characterized by preserved and even enhanced performance in executive function and crystallized intelligence. The impaired group in the current study and that of the Wenzel study showed significant underperformance compared to healthy individuals. The decrements in the impaired group were most pronounced in executive functioning. In terms of the spared cluster, both samples also identified individuals that seem to be performing within the normal range of cognition, and overperformance in cognitive flexibility, which falls within the domain of executive functioning. The current study further expands this field of research both methodologically and conceptually by testing the hypothesis that mechanisms related to accelerated aging, both at the global and local level may underpin the cognitive stratification of patients.

All prior studies in schizophrenia focused on sMRI-derived G-brainAGE (17–23), which has been found to be increased both at the early (18, 21) and more chronic disease stages (17, 19, 20, 22, 23). This study extends the investigation of G-brainAGE by providing evidence that increases in this metric may be confined to those patients who also manifest cognitive impairment. This proposition was also supported by the findings of L-brainAGE that showed a pattern of widespread apparent brain aging that was most pronounced in frontal and temporal regions. Notably, higher L-brainAGE was noted in brain regions where accelerated age-related decreases have been found in longitudinal studies of patients with schizophrenia (7, 8).

Patients in the spared cluster showed accelerated aging confined to the anterior cingulate cortex, which is crucial for integrating cognitive and emotional processes in support of goal directed behavior (41–44), and is often implicated in very early stages of psychosis (45–47). The degree of age-acceleration in the anterior cingulate cortex was small and it would appear that any dysfunction in this region in the spared cluster may not be of sufficient magnitude to lead to measurable cognitive difficulties.

The two clusters did not differ significantly in symptom severity or general functioning. Additionally, there was no association between brain-aging metrics and either of these domains suggesting some degree of specificity of the G-brainAGE and L-brainAGE findings to cognition. We also did not find evidence of an association between brain aging metrics and antipsychotic medication although patients in the impaired cluster had been exposed to higher doses of antipsychotic medication, as medication exposure is often driven by clinical severity, it is not possible to comment whether medication may have had an independent effect on our findings.

The current study has several limitations. Firstly, its cross-sectional nature does not allow inferences about the temporal association between brain aging and cognition. Further longitudinal studies are therefore important particularly in light of the report by Schnack et al. (19) that G-brainAGE in patients with schizophrenia increases further particularly during the first 2 years after disease onset. Second, the study does not address the evolution of cognitive function in schizophrenia and its possible impact on the long-term stability of the clusters identified here (2, 9). Third, the sample size of this study is modest. Larger sample sizes are needed to address generalizability and more robust identification of cognitive clusters and their associations with G-brainAGE and L-brainAGE, as well as with a wider range of clinical and behavioral features. Fourth, the current study does not inform on molecular changes that may underlie brain aging in schizophrenia. The available literature on brain aging suggests a potential role mainly for metabolic dysfunction (48) and low-level neuroinflammation (49) that should be assessed in future studies.

## Conclusion

In this study, we found differential patterns of accelerated brain aging in patients with schizophrenia based on their cognitive performance, with greater global accelerated brain aging, and more widespread local accelerated brain aging in patients with impaired than those with spared cognition. Further studies in longitudinal cohorts are needed to better understand progressive changes in accelerated aging and their association with the evolution of cognitive dysfunction and with molecular pathways involved in aging.

## Data Availability Statement

Publicly available datasets were analyzed in this study. This data can be found here: Human Connectome Project Young Adults (http://db.humanconnectome.org) and Human Connectome Project for Early Psychosis (https://nda.nih.gov/general-query.html?q=query=featureddatasets:Connectomes%20Related%20to%20Human%20Disease).

## Ethics Statement

The studies involving human participants were reviewed and approved by the Human Connectome Project. The patients/participants provided their written informed consent to participate in this study.

## Author Contributions

SH, RK, AR, HW, and SF contributed to conception and design of the study. SH organized the database and wrote the first draft of the manuscript. SH, RG, AM, and NS performed the statistical analysis. NS, SF, and RG wrote sections of the manuscript. All authors contributed to manuscript revision, read, and approved the submitted version.

## Funding

SF received support from the National Institute of Mental Health under grant R01MH113619 and the National Institute on Aging under grant R01AG058854.

## Conflict of Interest

The authors declare that the research was conducted in the absence of any commercial or financial relationships that could be construed as a potential conflict of interest.

## Publisher's Note

All claims expressed in this article are solely those of the authors and do not necessarily represent those of their affiliated organizations, or those of the publisher, the editors and the reviewers. Any product that may be evaluated in this article, or claim that may be made by its manufacturer, is not guaranteed or endorsed by the publisher.
