# Supplementary material for: Accelerated Global and Local Brain Aging Differentiate Cognitively Impaired From Cognitively Spared Patients With Schizophrenia
Source: Front Psychiatry. 2022 Jun 22;13:913470. doi: 10.3389/fpsyt.2022.913470 (PMC9257006; doi:10.3389/fpsyt.2022.913470)
Supplement: Supplementary file 1 [file Table_1.DOCX]

Supplementary Material

# Supplementary Methods

## Samples

### HCP-Early Psychosis

The Human Connectome Project-Early Psychosis study (HCP-EP) is an ongoing study aiming to recruit patients with schizophrenia (onset within 5 years of study entry) and demographically matched healthy individuals aged 16-35 years. The study has four recruitment sites: Indiana University, Beth Israel Deaconess Medical Center – Massachusetts Mental Health Center, McLean Hospital, and Massachusetts General Hospital. Healthy participants were recruited on the basis of the absence of lifetime diagnosis of psychotic, mood and anxiety disorders. Exclusion criteria for all participants included: 1) substance-induced psychosis; 2) IQ below 70 based on the Wechsler Abbreviated Scale of Intelligence, Second Edition (WASI-II); 3) medical condition that affects the brain or cognitive functioning (seizures, epilepsy, head trauma, stroke, traumatic brain injury, significant loss of consciousness, or other neurological disorder); 4) contraindication to undergo MRI scan; 5) severe substance use disorder in past 90 days; 6) electroconvulsive therapy in past 12 months; 7) risk for suicidal acts or any suicide attempt in past 30 days; 8) overtly aggressive behavior or who pose a substantial risk of danger.

### HCP-Young Adults

The Human Connectome Project – Young Adults (HCP-YA) recruits healthy individuals at the Washington University in St. Louis, Missouri. Participants have a Mini Mental Status Exam score of 29 or 30, and have no significant history of any psychiatric disorder, substance abuse, neurological, or cardiovascular disease as determined by: 1) no hospitalization; 2) no report of diagnosis by a treating physician; 3) no pharmacologic or behavioral treatment by a physician or therapist. Exclusion criteria included: 1) two or more seizures or a diagnosis of epilepsy; 2) genetic disorder; 3) use of prescription medication in past 12 months; 4) multiple sclerosis; 5) cerebral palsy; 6) brain tumor; 7) stroke; 8) sickle cell disease; 9) thyroid hormone treatment in past 12 months; 10) current chemotherapy or history of radiation or chemotherapy that could affect the brain; 11) current treatment for diabetes; 12) head injury with loss of consciousness greater than 30 minutes; 13) premature birth; 14) pregnancy for females; 15) unsafe metal or devices in the body; and 16) claustrophobia.

## Cognitive Clustering

### HYDRA

This algorithm is a non-linear machine learning algorithm for integrated binary classification and subpopulation clustering (1). Key advantages of HYDRA, over other machine learning techniques, are that it disposes of the need for a priori specification of the number of clusters and does not use similarity measures for clustering as such measures are susceptible to the effect of non-specific factors such as age and sex. The detailed code can be found on https://github.com/evarol/HYDRA. Classification in HYDRA is based on indices of deviation between a clinical and the healthy reference group; healthy individuals are separated from the clinical sample using a convex polytope formed by combining multiple linear hyperplanes. This confers an additional advantage to HYDRA because the multiple hyperplanes model potential heterogeneity within clinical samples while their combination extends linear max-margin classifiers to the non-linear space.

### K-means Clustering

The k-means algorithm implemented in R (version 4.1.0) using the NbClust package (version 3.0) was applied to the same cognitive variables as the HYDRA algorithm to test for the algorithm independence in reproducibility. In the algorithm, K points, serving as initial cluster centroids, are placed into the space represented by the sample. Then each participant is assigned to a cluster based on its proximity to its centroid. When all participants are assigned, the positions of the K centroids are recalculated. The process is then repeated until the centroids remain unchanged. This process separates participants into homogenous clusters while maximizing heterogeneity between clusters. The optimal number of clusters is identified based on the solution endorsed by majority vote of fit indices. The clusters of the optimal solution were compared with the clusters identified using the HYDRA algorithm in terms of cognitive profiles within each of the clusters and overlap of cluster solutions across the two methods.

## Neuroimaging

### Acquisition

#### HCP-EP

In the HCP-EP sample, T1-weighted images were acquired on Siemens MAGNETOM Prisma 3T scanners using a 3D magnetization-prepared rapid gradient-echo (MPRAGE) sequence from 3 sites: Brigham and Women’s Hospital (BWH), McLean Hospital, and at Indiana University (IU). The BWH and IU sites used a 32-channel head coil. The McLean used a 64-channel head & neck coil, with the neck channels turned off. All sites used the following parameters: repetition time (TR)/time to echo (TE)/inversion time (TI)=2400/2.22/1000 ms, voxel size=0.8 mm isotropic, flip angle=8°, field of view (FOV)=256 × 256 mm2, duration of acquisition: 6 mins 38s.

#### HCP-Young Adults

In the HCP-YA sample, T1-weighted images were acquired on a 3T Siemens Skyra scanner using a 3D MPRAGE sequence with the following parameters: TR/TE/TI= 2400/2.14/1000 ms, voxel size = 0.7 mm isotropic, flip angle = 8°, FOV = 224 × 224 mm2, duration of acquisition: 7 min 40 s.

### Preprocessing

Preprocessing of HCP-YA and HCP-EP neuroimaging data was carried out using standard pipelines implemented in the Statistical Parametric Mapping (SPM12) software package (https://www.fil.ion.ucl.ac.uk/spm/software/spm12/) to derive input features for global and local brain age prediction as described below. Brain tissue was segmented into grey matter (GM) and white matter (WM) maps which were registered to the Montreal Neurological Institute 152 (MNI152) space using a nonlinear registration procedure implemented with DARTEL algorithm (2), and were then resampled to 1.5 mm^3^ with a 4mm smoothing kernel.

## Local and Global brainAGE Computation

We calculated L-brainAGE using the methods developed by Popescu and colleagues (3). Specifically they collated data from multiple cohorts to create a discovery sample comprising 3463 T1-weighted MRI brain scans from healthy people (aged 18-90 years) while an independent sample of 692 healthy people was used as the hold-out test sample. Data from the discovery sample were randomly subsampled and split into a training (80%) and validation set (20%). The model parameters were determined in the training set and applied to the validation set. The training process involved entering the modulated GM and WM maps derived from SPM12 as input features to a convolutional neural network (CNN) that adopted by the U-Net architecture introduced by Ronneberger and colleagues (4) (<https://github.com/SebastianPopescu/U-NET-for-LocalBrainAge-prediction>). The GM and WM maps images were split into overlapping 3-dimensional blocks of 523 voxels. The convolutional layers in the network used an isotropic 3×3×3 filter, convolved over the input image after which element-wise multiplication with the filter weights and subsequent summation was performed at each location. Subsequently, to allow for non-linear modelling, the obtained values were processed using an “activation function”; specifically, an activation function (i.e., LeakyReLu) with α=0.2 was used. LeakyReLu(α) was defined as: LeakyReLu(x) = max(x, 0) + min(x * α, 0), which allows a small, non-zero gradient when the unit is not “active”. The convolution operation was also controlled by its stride, which is how many voxels are skipped after every element-wise weight multiplication and summation. The value of stride was set to 1. The algorithm used down-sampling which increases the effective field of view or “receptive field” of layers higher in the hierarchy. Down-sampling at each scale was implemented using two consecutive 3D 3×3×3 filter kernels with an initial number of channels set to 64, which was multiplied by 2 further down the down-sampling path. Down-sampling involved 2×2×2 average pooling. For the up-sampling part of the network, the down-sampling architecture was inverted, by replacing the down-sampling layers with 2×2×2 up-sampling layers. Each convolution used a squeeze-and-excite unit based on the Squeeze & Excite networks (5) to obtain age predictions over 123 voxels blocks. Voxel-level mean absolute error (MAE) cost function on the output layer and two additional cost functions at the two other scales of the architecture were used: global average pooling followed by a dense layer to predict brain-age at block-level. The model was implemented in TensorFlow (6).

Neuroimaging-based age-prediction is subject to regression dilution leading to a greater under- or over-estimate of age, the further away a sample is from the training set mean age. To account for this effect, Popescu et al used separate small batch (n=200) of participants randomly selected from the held-out dataset and obtained their voxel-level brain-age delta ∆_(i,v) (i.e., predicted minus actual age), where i indicates the i-th participant and v the v-th voxel. Then participants were grouped according to their chronological aged into 5-year bins, with the first bin covering participants with a chronological age between 18-25 year. For each bin b the corresponding average voxel-level brain-age delta ∆_(b,v) was calculated, which represents the average brain-age delta for that voxel given the chronological age interval. Subsequently, to de-bias the voxel-level brain-age delta for a new participant (e.g., from testing set), ∆_(j,v) the following formula was used:

〖∆_(j,v)^~ ∆〗_(j,v) 〖-∆〗_(b,v)

The accuracy of the model parameters from the discovery dataset was then tested in the hold-out sample (n=692). The voxel-level MAE (unadjusted) values of the model varied in different brain regions; lower values were in the prefrontal cortex and subcortical regions and higher in the occipital lobe, cerebellum and brainstem.

The model developed by Popescu and colleagues was applied to the preprocessed voxel-based maps of the HCP-YA and HCP-EP samples using the same procedures described above. This resulted in individualized voxel-based maps of L-brainAGE distributions across the brain. L-brainAGE voxel-based maps were read into Matlab 2019a using the built-in niftiread function and subsequently the mean of the L-brainAGE voxel-based maps was computed, while excluding voxels outside the brain and within ventricles using a mask, to yield individualized G-brainAGE scores. G-brainAGE estimates were corrected for any residual effects of age as per Le et al. (7), and the corrected estimates were used in all subsequent analyses unless otherwise specified.

# Supplementary Results

## K-means Clustering Cognitive Profiles

K-means clustering identified two clusters with a majority vote of 10 fit indices (while other solutions were supported by 3 or less). Individuals in cluster 1 (n = 66) showed a similar pattern of cognitive decrement across cognitive tests, while individuals in cluster 2 (n=18) showed generally intact cognition (Supplementary Figure 1), in line with the findings using the HYDRA clustering technique. Across both clustering methods, we found an 89.29% overlap in patients assigned to the impaired and spared cluster identified using the two methods. Specifically, three patients from the spared HYDRA cluster were identified as impaired in the K-means clusters. Examination of the individual profiles of these three patients showed that they were more impaired in working memory (n=1), and emotional processing (n=2). Six patients from the impaired HYDRA cluster were identified as spared in the K-means clusters. Examination of the individual profiles of these six patients showed that they were less impaired in crystallized intelligence (n=6).

## Mean Absolute Error (MAE) in the L-brainAGE model

Local MAE values showed a ventral to dorsal and a posterior to anterior gradient of decreasing values in healthy individuals, ranging from 3.36 to 16.67, with the greatest MAE in the cerebellum (Supplementary Figures 2 and 3).

## Association between G-brainAGE and chronological age

There was no residual association between chronological age and G-brainAGE corrected for age-bias across both samples of healthy individuals and patients (Supplementary Figure 4).

## Univariate associations of predictors with G-brainAGE across all patients

In the patient group, there were no significant associations between G-brainAGE and PANSS scores, antipsychotic medication exposure, occupational and social functioning, and WASI-II IQ (Supplementary Table 4).

# Supplementary Figures and Tables

##
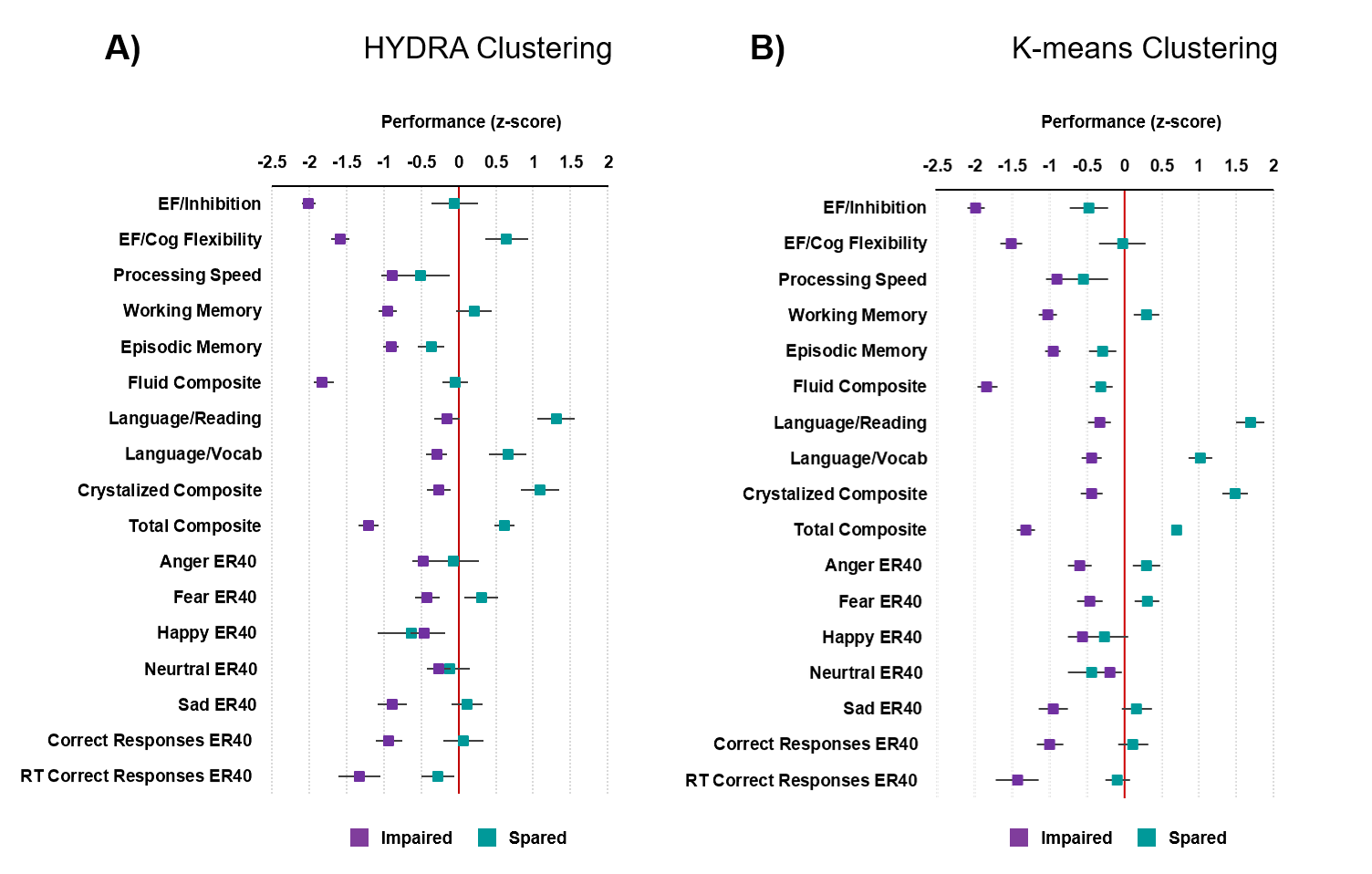
Supplementary Figures


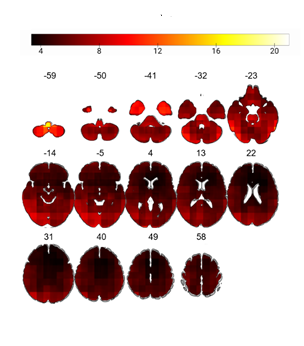
**Supplementary Figure 1. Comparison of cognitive profiles of patients with schizophrenia using two clustering methods.** Average standardized cognitive performance profiles in the impaired and spared cognitive subgroups identified using HYDRA and K-means clustering. Error bars represent standard error. For RT Correct Responses ER40, the directionality of values was reversed so that higher values denote better performance. Executive Function/Inhibition: NIH Toolbox Flanker Inhibitory Control and Attention Test; Executive Function (EF)/Cognitive Flexibility: NIH Toolbox Dimensional Change Card Sort Test; Processing Speed: NIH Toolbox Pattern Comparison Processing Speed Test; Working Memory: NIH Toolbox List Sorting Working Memory Test; Episodic Memory: NIH Toolbox Picture Sequence Memory Test; Fluid IQ: NIH Toolbox Cognition Fluid Composite; Language/Reading: NIH Toolbox Oral Reading Recognition Test; Language/Vocabulary: NIH Toolbox Picture Vocabulary Test; Crystalized IQ: NIH Toolbox Cognition Crystallized Composite; Full IQ: NIH Toolbox Cognition Total Composite Score; Penn Emotion Recognition Test: Number of Correct Anger Identifications: Anger ER40; Penn Emotion Recognition Test: Number of Correct Fear Identifications: Fear ER40; Penn Emotion Recognition Test: Number of Correct Happy Identifications: Happy ER40; Penn Emotion Recognition Test: Number of Correct Neutral Identifications: Neutral ER40; Penn Emotion Recognition Test: Number of Correct Sad Identifications: Sad ER40; Penn Emotion Recognition Test Number of Correct Responses: Correct ER40: Penn Emotion Recognition Test: Correct Responses Median Response Time: RT Correct Responses ER40.

**
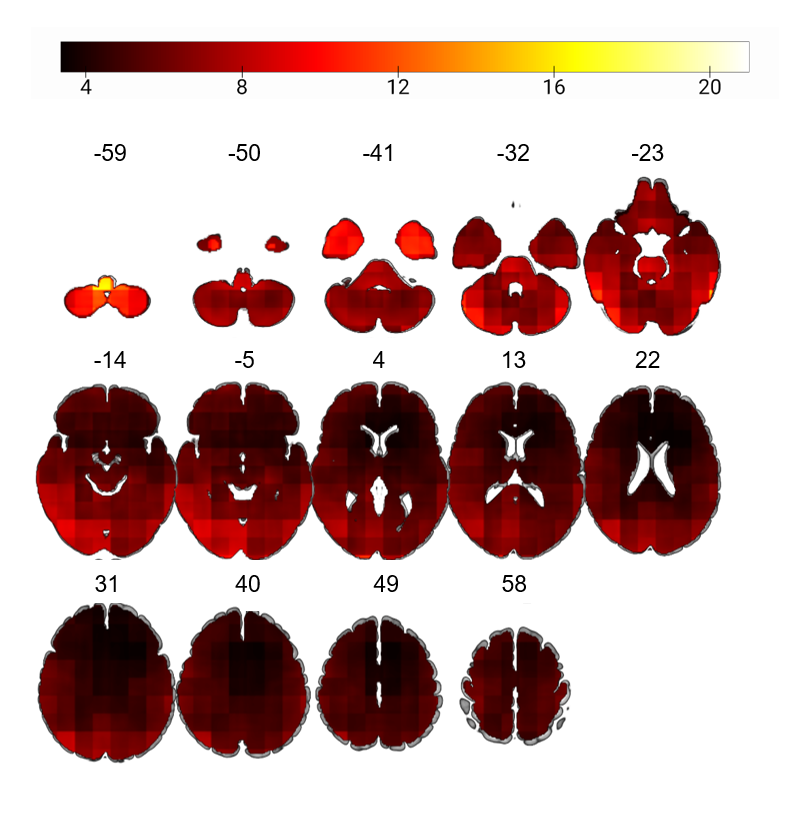
**

**Supplementary Figure 2. Spatial distribution of MAE in the L-brainAGE model in healthy individuals.**


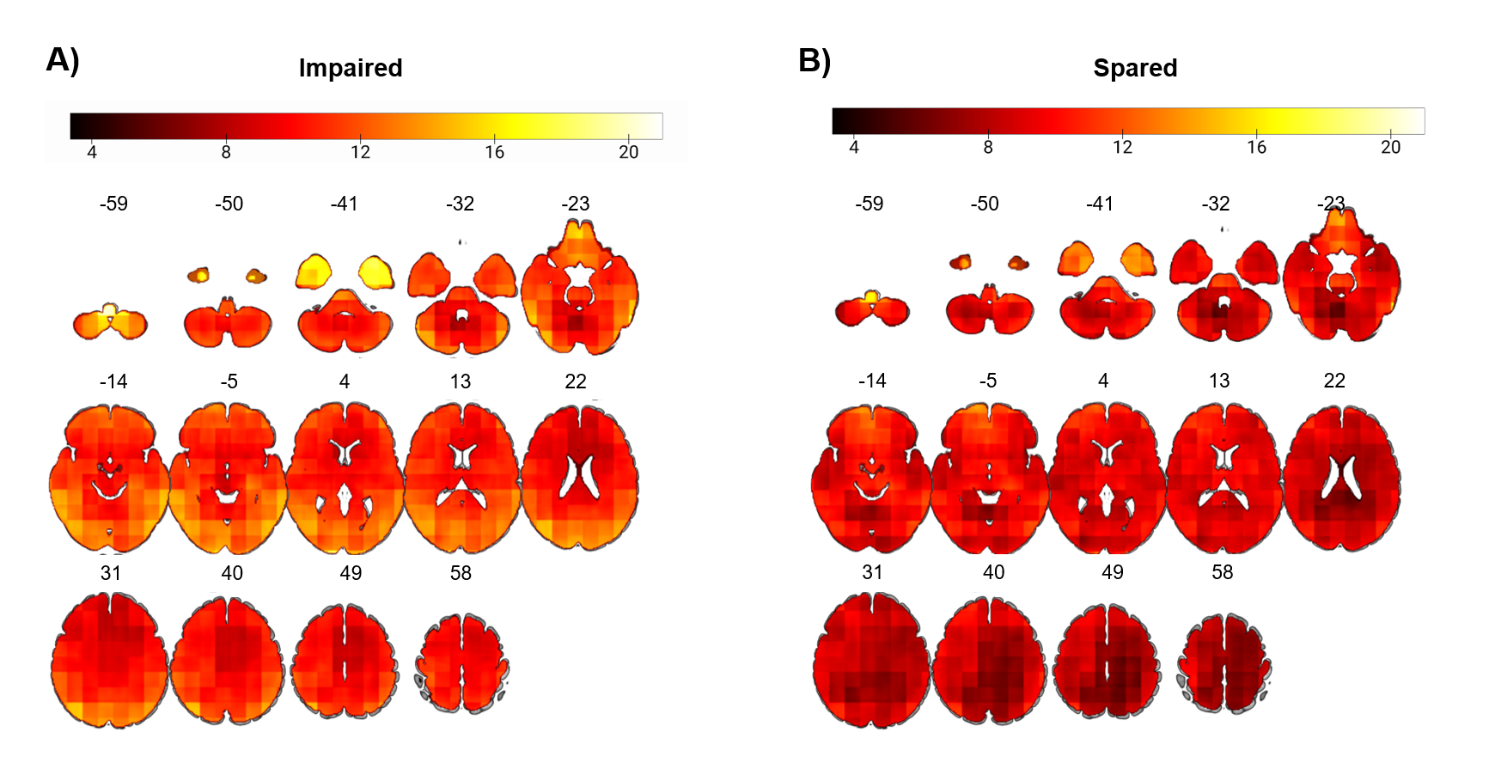


**Supplementary Figure 3. Spatial distribution of MAE in the L-brainAGE model in the impaired and spared cluster.**


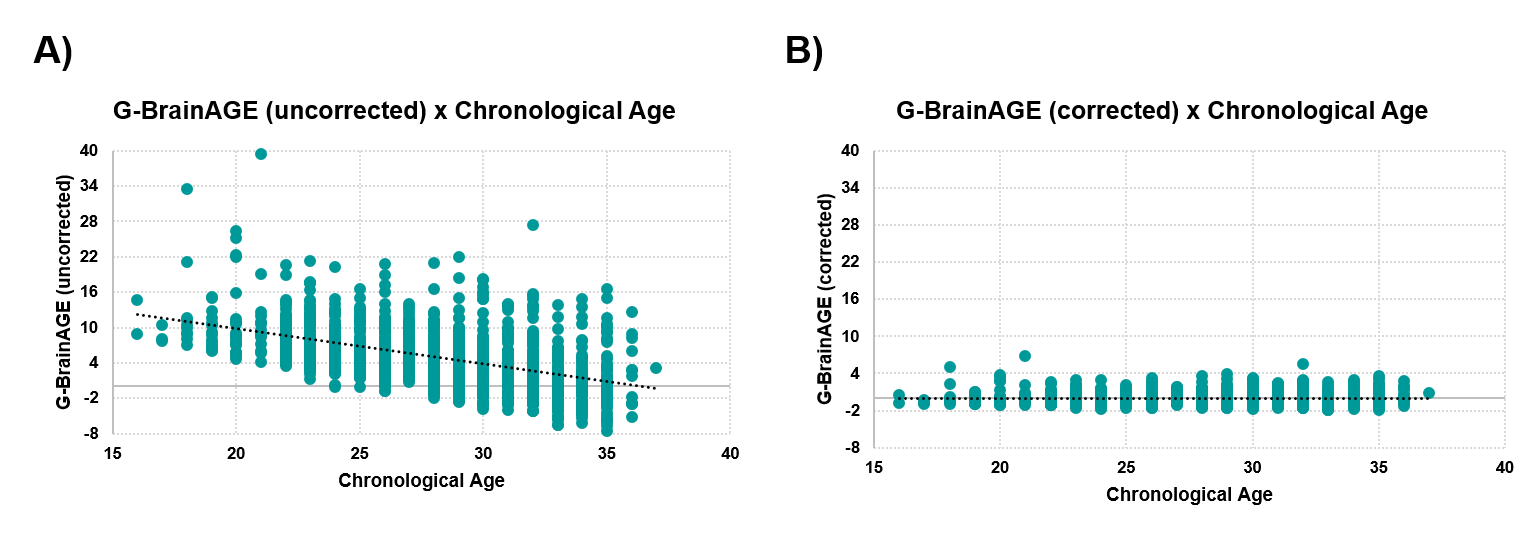


**Supplementary Figure 4. Correlation between chronological and G-brainAGE in the entire study sample.** (A) Scatter plot illustrating negative correlations between uncorrected G-brainAGE and chronological age; (B) Scatter plot illustrating absence of residual associations between corrected G-brainAGE and chronological age.

## Supplementary Tables

| **Supplementary Table 1. Sample Characteristics** | | | |
| --- | --- | --- | --- |
|  | **Patients**  **(N = 84)** | **Healthy Individuals**  **(N = 1169)** | **T/χ^2^** |
| **Sociodemographic** | | | |
| Degrees & credentials earned, mean (SD) | 6.02 (2.06) | 8.66 (2.30) | -8.20*** |
| Years of education, mean (SD) | 13.59 (2.01) | 14.91 (1.91) | -4.67*** |
| **Clinical** | | | |
| Antipsychotic naïve, N (%) | 4 (3.17%) | - | - |
| Antipsychotic Dose (CPZE), mean (SD) | 178.97 (229.81) | - | - |
| Antipsychotic Drug Exposure (months), mean (SD) | 14.83 (15.32) | - | - |
| PANSS Positive, mean (SD) | 12.30 (4.61) | - | - |
| PANSS Negative, mean (SD) | 14.06 (5.48) | - | - |
| MIRECC-GAF Occupational Functioning | 64.46 (23.42) | 89.46 (13.24) | -8.12*** |
| MIRECC-GAF Social Functioning | 68.54 (16.20) | 87.10 (15.14) | -7.79*** |
| **Cognition** |  |  |  |
| Episodic Memory, mean (SD) | 89.39 (12.80) | 105.08 (16.43) | -10.03*** |
| Executive Function/Cognitive Flexibility, mean (SD) | 83.26 (19.59) | 102.12 (10.48) | -16.77*** |
| Executive Function/Inhibition, mean (SD) | 75.79 (16.85) | 101.09 (10.63) | -23.03*** |
| Language/Reading Decoding, mean (SD) | 102.76 (21.13) | 107.29 (15.16) | -2.96** |
| Language/Vocabulary Comprehension, mean (SD) | 99.93 (17.96) | 109.30 (15.44) | -6.16*** |
| Processing Speed, mean (SD) | 89.55 (19.49) | 103.59 (19.97) | -7.27*** |
| Working Memory, mean (SD) | 89.83 (16.17) | 103.21 (13.31) | -10.15*** |
| Cognition Fluid Composite, mean (SD) | 78.95 (16.49) | 105.29 (17.06) | -15.97*** |
| Cognition Crystallized Composite, mean (SD) | 101.24 (20.21) | 110.16 (17.14) | -5.28*** |
| Cognition Total Composite Score, mean (SD) | 88.61 (18.65) | 113.01 (20.25) | -12.51*** |
| **Emotion Recognition** | | | |
| Penn Emotion Recognition Test: Correct Responses, mean (SD) | 33.92 (3.34) | 35.58 (2.51) | -6.60*** |
| Penn Emotion Recognition Test: Correct Responses Median Response Time (ms), mean (SD) | 2244.46 (777.53) | 1826.98 (329.03) | 11.01*** |
| Penn Emotion Recognition Test: Correct Anger Identifications, mean (SD) | 6.43 (1.16) | 6.77 (1.02) | -3.37*** |
| Penn Emotion Recognition Test: Correct Fear Identifications, mean (SD) | 6.70 (1.36) | 6.93 (1.16) | -1.97* |
| Penn Emotion Recognition Test: Correct Happy Identifications, mean (SD) | 7.87 (0.34) | 7.96 (0.22) | -3.76*** |
| Penn Emotion Recognition Test: Correct Neutral Identifications, mean (SD) | 7.07 (1.46) | 7.15 (1.23) | -0.68 |
| Penn Emotion Recognition Test: Correct Sad Identifications, mean (SD) | 6.09 (1.62) | 6.79 (1.13) | -6.12*** |
| MIRECC-GAF = Mental Illness Research, Education, and Clinical Center version of the Global Assessment of Functioning scale; CPZE=Chlorpromazine Equivalents; PANSS = Positive and Negative Syndrome Scale; SD = standard deviation; significance levels: **P*<0.05; ***P*<0.01; ****P*<0.001 | | | |
| . | | | |

| **Supplementary Table 2. Definition of the cognitive variables used for clustering** | | |
| --- | --- | --- |
| **Domain** | **Variable Name** | **Description** |
| **NIH Toolbox** | | |
| Episodic Memory | PicSeq_AgeAdj | NIH Toolbox Picture Sequence Memory Test: Age-Adjusted Scale Score |
| Executive Function/Cognitive Flexibility | CardSort_AgeAdj | NIH Toolbox Dimensional Change Card Sort Test: Age-Adjusted Scale Score |
| Executive Function/Inhibition | Flanker_AgeAdj | NIH Toolbox Flanker Inhibitory Control and Attention Test: Age-Adjusted Scale Score |
| Language/Reading Decoding | ReadEng_AgeAdj | NIH Toolbox Oral Reading Recognition Test: Age-Adjusted Scale Score |
| Language/Vocabulary Comprehension | PicVocab_AgeAdj | NIH Toolbox Picture Vocabulary Test: Age-Adjusted Scale Score |
| Processing Speed | ProcSpeed_AgeAdj | NIH Toolbox Pattern Comparison Processing Speed Test: Age-Adjusted Scale Score |
| Working Memory | ListSort_AgeAdj | NIH Toolbox List Sorting Working Memory Test: Age-Adjusted Scale Score |
| Cognition Fluid Composite | CogFluidComp_AgeAdj | NIH Toolbox Cognition Fluid Composite: Age Adjusted Scale Score |
| Cognition Crystallized Composite | CogCrystalComp_AgeAdj | NIH Toolbox Cognition Crystallized Composite: Age Adjusted Scale Score |
| Cognition Total Composite Score | CogTotalComp_AgeAdj | NIH Toolbox Cognition Total Composite Score: Age Adjusted Scale Score |
| **Penn Emotion Recognition Test** | | |
| Emotion Recognition | ER40_CR | Penn Emotion Recognition Test: Number of Correct Responses |
| Emotion Recognition | ER40_CRT | Penn Emotion Recognition Test: Correct Responses Median Response Time (ms) |
| Emotion Recognition | ER40ANG | Penn Emotion Recognition Test: Number of Correct Anger Identifications |
| Emotion Recognition | ER40FEAR | Penn Emotion Recognition Test: Number of Correct Fear Identifications |
| Emotion Recognition | ER40HAP | Penn Emotion Recognition Test: Number of Correct Happy Identifications |
| Emotion Recognition | ER40NOE | Penn Emotion Recognition Test: Number of Correct Neutral Identifications |
| Emotion Recognition | ER40SAD | Penn Emotion Recognition Test: Number of Correct Sad Identifications |

| **Supplementary Table 3. Cognitive performance of the impaired and spared cognitive clusters** | | | |
| --- | --- | --- | --- |
| **Measure** | **Impaired (N = 69)** | **Spared (N = 15)** | **T/χ^2^ (p-value)** |
| **Cognition** | | | |
| Episodic Memory, mean (SD) | 86.41 (13.10) | 94.53 (10.28) | **-2.25 (0.03)** |
| Executive Function/Cognitive Flexibility, mean (SD) | 76.16 (15.53) | 109.67 (16.5) | **-7.49 (<0.001)** |
| Executive Function/Inhibition, mean (SD) | 69.87 (11.56) | 99.20 (18.17) | **-7.83 (<0.001)** |
| Language/Reading Decoding, mean (SD) | 97.62 (20.93) | 119.60 (14.47) | **-3.86 (<0.001)** |
| Language/Vocabulary Comprehension, mean (SD) | 95.55 (17.42) | 109.87 (14.52) | **-2.96 (0.004)** |
| Processing Speed, mean (SD) | 86.67 (18.26) | 92.40 (22.57) | -1.06 (0.29) |
| Working Memory, mean (SD) | 85.78 (15.28) | 103.13 (13.72) | **-4.05 (<0.001)** |
| Cognition Fluid Composite, mean (SD) | 72.56 (13.75) | 99.33 (9.83) | **-7.14 (<0.001)** |
| Cognition Crystallized Composite, mean (SD) | 95.95 (19.57) | 116.34 (14.85) | **-3.80 (<0.001)** |
| Cognition Total Composite Score, mean (SD) | 81.85 (16.55) | 109.20 (7.82) | **-6.23 (<0.001)** |
| **Emotion Recognition** | | | |
| Penn Emotion Recognition Test: Correct Responses, mean (SD) | 32.97 (3.76) | 35.60 (2.77) | **-2.56 (0.01)** |
| Penn Emotion Recognition Test: Correct Responses Median Response Time (ms), mean (SD) | 2408.78 (957.75) | 1979.53 (343.79) | 1.71 (0.09) |
| Penn Emotion Recognition Test: Correct Anger Identifications, mean (SD) | 6.25 (1.29) | 6.67 (1.35) | -1.14 (0.26) |
| Penn Emotion Recognition Test: Correct Fear Identifications, mean (SD) | 6.41 (1.59) | 7.27 (1.03) | **-2.00 (0.05)** |
| Penn Emotion Recognition Test: Correct Happy Identifications, mean (SD) | 7.84 (0.37) | 7.80 (0.41) | 0.38 (0.71) |
| Penn Emotion Recognition Test: Correct Neutral Identifications, mean (SD) | 6.81 (1.66) | 7.00 (1.31) | -0.41 (0.68) |
| Penn Emotion Recognition Test: Correct Sad Identifications, mean (SD) | 5.67 (1.91) | 6.87 (0.99) | **-2.36 (0.02)** |
| SD=standard deviation | | | |
|  | | | |
|  | | | |

| **Supplementary Table 4. Spearman’s correlation coefficients between G-brainAGE and clinical variables in patients** | | | |
| --- | --- | --- | --- |
| **Measure** | **Impaired Cluster**  **(N = 69)** | **Spared Cluster**  **(N = 15)** | **All Patients**  **(N = 84)** |
| **PANSS Positive Symptoms** | 0.11 | 0.48 | 0.16 |
| **Antipsychotic Drug Dose (CPZE)^a^** | 0.05 | 0.03 | -0.003 |
| **MIRECC-GAF Occupational Functioning** | -0.01 | -0.38 | -0.05 |
| **MIRECC-GAF Social Functioning** | -0.04 | -0.02 | -0.04 |
| **WASI-II IQ** | -0.09 | -0.40 | -0.13 |
| ^a^Medication information was only available in subjects recruited at Indiana Univeristy. MIRECC-GAF = Mental Illness Research, Education, and Clinical Center version of the Global Assessment of Functioning scale; WASI-II IQ = Wechsler Abbreviated Scale of Intelligence, Second Edition; PANSS = Positive and Negative Syndrome Scale; all pairwise *P*> 0.10 | | | |
|  | | | |

# References

1. Varol E, Sotiras A, Davatzikos C, Initiative AsDN. Hydra: Revealing Heterogeneity of Imaging and Genetic Patterns through a Multiple Max-Margin Discriminative Analysis Framework. *Neuroimage* (2017) 145:346-64. doi: 10.1016/j.neuroimage.2016.02.041

2. Ashburner J. A Fast Diffeomorphic Image Registration Algorithm. *Neuroimage* (2007) 38(1):95-113. Epub 2007/09/01. doi: 10.1016/j.neuroimage.2007.07.007.

3. Popescu SG, Glocker B, Sharp DJ, Cole JH. Local Brain-Age: A U-Net Model. *Front Aging Neurosci* (2021) 13:761954. Epub 2021/12/31. doi: 10.3389/fnagi.2021.761954.

4. Ronneberger O, Fischer P, Brox T, editors. U-Net: Convolutional Networks for Biomedical Image Segmentation. *International Conference on Medical image computing and computer-assisted intervention*; 2015: Springer.

5. Hu J, Shen L, Sun G, editors. Squeeze-and-Excitation Networks. *Proceedings of the IEEE conference on computer vision and pattern recognition*; 2018. doi:10.1109/TPAMI.2019.2913372

6. Abadi M, Barham P, Chen J, Chen Z, Davis A, Dean J, et al., editors. {Tensorflow}: A System for {Large-Scale} Machine Learning. *12th USENIX symposium on operating systems design and implementation (OSDI 16)*; 2016.

7. Le TT, Kuplicki RT, McKinney BA, Yeh H-W, Thompson WK, Paulus MP, et al. A Nonlinear Simulation Framework Supports Adjusting for Age When Analyzing Brainage. *Frontiers in aging neuroscience* (2018) 10:317. doi: 10.3389/fnagi.2018.00317
